# Papillary renal cell carcinoma, formerly known as Type 2: a single institutional study addressing histologic and molecular features

**DOI:** 10.1111/his.70069

**Published:** 2025-12-16

**Authors:** Melissa Yuwono Tjota, Jung Woo Kwon, Pankhuri Wanjari, Tatjana Antic

**Affiliations:** ^1^ Department of Pathology The University of Chicago Chicago IL USA

**Keywords:** eosinophilic cytoplasm, molecular heterogeneity, papillary renal cell carcinoma

## Abstract

**Aims:**

Papillary renal cell carcinoma (pRCC) accounts for 15%–20% of RCC cases and is the second most common histologic subtype of RCC. In contrast to other common RCC subtypes, there continues to be ongoing debate about how to classify RCCs with papillary architecture and eosinophilic cytoplasm given the heterogeneity of histologic, IHC and molecular findings. Our study set out to characterize the histologic features and molecular alterations in cases that were originally diagnosed as pRCC, Type 2 or high‐grade pRCC in a single institutional study (*n* = 63).

**Methods and results:**

Histologically, the vast majority of the cases had a papillary pattern (*n* = 59). There were three cases that had a mixed solid and papillary pattern and one case that had a sarcomatoid architectural pattern. The cases were composed of large cells with eosinophilic cytoplasm, pseudostratified or apically oriented nuclei and prominent nucleoli. Molecular analysis of these cases revealed a wide range of genes that were mutated, with the most common ones being *SETD2* (*n* = 9), *PBRM1* (*n* = 5), *KDM6A* (*n* = 7), *PMS2* (*n* = 4), *NF2* (*n* = 7) and *TERT* (*n* = 7). Our study further identified cases that had molecular mutations in the RTK/RAS pathway (*KRAS* and *NRAS*), PI3K pathway (*TSC2*), TP53 pathway (*TP53* and *CHEK2*) and copy number alterations in the cell cycle pathway (*CDKN2A* and *CCND3*).

**Conclusions:**

These findings highlight the need to molecularly characterize these lesions as there is no specific histologic finding to identify cases that harbour different pathogenic alterations in specific genes.

## Graphical abstract

Papillary renal cell carcinoma (pRCC) accounts for 15%–20% of RCC cases and is the second most common histologic subtype of RCC. In contrast to other common RCC subtypes, there continues to be ongoing debate about how to classify RCCs with papillary architecture and eosinophilic cytoplasm, given the heterogeneity of histologic, IHC and molecular findings. Our goal with this study was to further characterize histologic features and molecular alterations in sporadic cases that were originally diagnosed as pRCC, Type 2 or high‐grade pRCC in a single institutional study.

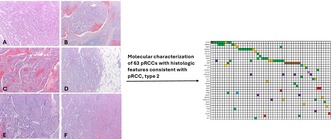

**Abbreviations**
AJCC: American Joint Committee on Cancer
ccRCC: clear cell RCC
IGV: integrated genomics viewer
ISUP: International Society of Urological Pathology
pRCC: papillary renal cell carcinoma
PRNRP: papillary renal neoplasm with reverse nuclear polarity
WHO: World Health Organization

## Introduction

Papillary renal cell carcinoma (pRCC) accounts for 15%–20% of RCC cases and is the second most common histologic subtype of RCC.^1^ The 2016 WHO subclassified pRCC into pRCC, Type 1 (scant basophilic cytoplasm with WHO/ISUP grade 1 and 2) and pRCC, Type 2 (eosinophilic cytoplasm with WHO/ISUP nuclear grade 3 and 4),^2^ which were shown to have significantly different clinical behaviour and prognosis.^3^ In 2022, the WHO changed its recommendation to diagnose cases as pRCC without further subclassification.^4^ This modification came about as the concept of two morphologically distinct subtypes has been challenged over the years with studies suggesting that 25% of cases have classical pRCC, Type 1 features, 25% of cases have classical pRCC, Type 2 features and 50% have some degree of overlap between pRCC, Type 1 and pRCC, Type 2 features.^5^

Since 1997, with the publication of ‘The Heidelberg classification of renal tumors’, there has been increasing integration of molecular findings into the diagnosis of renal tumours, most recently with the 2022 World Health Organization (WHO) tumour classification^4^ and the 2020 consensus report from the International Society of Urological Pathology (ISUP).^6^ Clear cell RCC (ccRCC), which accounts for 75% of RCCs, has been the best studied and is associated with mutations in *VHL* and loss of heterozygosity of chromosome 3p^7^. More studies that are recent have also shown alterations in *PBRM1*, *SETD2* and *BAP1*.^8^ Sporadic classical pRCC frequently demonstrates polysomy or trisomy of chromosomes 7 or 17. Studies have also seen gains of chromosome 3, 12, 16 and 20, as well as various chromosomal losses.^9^ Hereditary pRCC syndrome has been associated with *MET* mutations.^10^ Chromophobe RCC (CHRCC) has been shown to harbour numerous chromosomal losses (chromosomes 1, 2, 6, 10, 13, 17 and 21)^11^ as well as pathogenic mutations in *PTEN*, *TP53* and *TERT*.^11^, ^12^ A ‘histo‐molecular’ approach for defining renal tumours has been proposed, particularly as the 2022 WHO classification introduced molecularly defined renal carcinomas, including *TFE3/TFEB* rearranged/altered RCC, *ELOC* (formerly known as *TCEB1*) mutated RCC, *FH*‐deficient RCC, succinate dehydrogenase‐deficient RCC, *ALK‐*rearranged RCC and *SMARCB1*‐deficient medullary RCC.^4^

In contrast to other subtypes of RCC that have characteristic molecular alterations, studies have shown that pRCC, particularly cases with eosinophilic cytoplasm, is a heterogeneous group with recurrent mutations in *NF2, SETD2, BAP1, PBRM1, CDKN2A, TERT, TSC1, TSC2, MTOR* and other genes.^13^ Our goal with this study was to further characterize histologic features and molecular alterations in sporadic cases that were originally diagnosed as pRCC, type 2 or high‐grade pRCC in a single institutional study.

## Methods

### Demographics and Clinicopathologic Analysis

We retrospectively reviewed the Department of Pathology database from 2005 to 2018 and pulled all cases that had been previously diagnosed as pRCC, Type 2 or pRCC, high‐grade and were greater than 1.5 cm in size. This study only included cases that were available in‐house. The cases were reviewed by two genitourinary pathologists, and only cases that had papillary architecture were included for further studies. This resulted in 295 cases, of which 230 cases were excluded as they had morphology consistent with what was historically diagnosed as classic pRCC, Type 1. Out of the remaining 65 cases, two were excluded from the study as molecular sequencing performed for clinical management had demonstrated that one case harboured a pathogenic *FH* mutation in a patient that had a germline mutation and another case identified a pathogenic *FLCN* mutation. The remaining 63 (21.5%) cases were used for further investigation. Of note, 7 cases within this cohort had morphologic findings that would have included translocation RCC in the differential diagnosis. IHC work‐up during the clinical diagnosis of the case excluded translocation RCC as the cases were strongly and diffusely positive for keratins, including Cam5.2, AE1/AE3 and CK7 and/or negative for TFE3. High nuclear grade was defined as enlarged nuclei with prominent nucleoli and/or at least focally WHO/ISUP grade 3. Clinical data was obtained through electronic medical record review, including age and sex. This study was approved by the Institutional Review Board.

### Next‐Generation Sequencing

A representative formalin‐fixed, paraffin‐embedded block was selected for NGS on the University of Chicago Medicine OncoPlus (UCM‐OncoPlus) panel, a hybrid‐capture panel targeting 1005 cancer‐associated genes of which 168 genes are clinically reported. DNA extraction, DNA quantification, library preparation and NGS were performed as described previously.^14^ RNA sequencing was performed with the University of Chicago Medicine RNA Oncoplus assay. In addition to whole exon coverage of 1006 cancer‐associated genes, the panel is capable of detecting known and novel fusions involving any of the 1006 genes. RNA was isolated using the FFPE RNA Extraction Kit (Qiagen, Hilden, Germany). Library preparation was performed using adapter molecules with patient‐specific index sequences (KAPA Stranded RNA‐Seq Kit with RiboErase, Kapa Biosystems, Wilmington, MA). The libraries for DNA and RNA analysis were sequenced in a rapid run mode on a NovaSeq 6000 system (Illumina, San Diego, CA). Data analysis was performed on a high‐performance computing system (Center for Research Informatics, University of Chicago) using an in‐house developed bioinformatics pipeline, using the hg19 (GRCh37) human genome reference sequence for alignment. CNV Kit14 software with additional in‐house intra‐run normalization with comparison to a pooled cohort of clinical controls was used to calculate copy number results. Somatic variant calls were inspected using Integrated Genomics Viewer (IGV; Broad Institute, MIT Harvard, Cambridge, MA). Cosmic and ClinVar were used as additional tools to research and confirm detected variants.

## Results

### Clinical Demographics

Review of the H&E for 293 pRCCs resulted in the selection of 63 (21.5%) cases used for further investigation based on the histologic features described below. The patients ranged in age from 40 to 88 years (mean—64.4 years). Forty‐three patients were male (68.3%), and 20 patients were female (31.7%) (Table 1). None of the patients had a known history suggestive of hereditary disease.

**Table 1:** Demographics and clinicopathologic analysis

Demographics	
Age, y (SD)	64.4 (12.6)
Male, *n* (%)	43 (68.3)
Pathologic characteristics	
Tumour size, cm (SD)	5.6 (4.1)
High‐grade nuclear features, *n* (%)	30 (47.6)
Architectural pattern	
Papillary, *n* (%)	59 (93.7)
Solid and papillary, *n* (%)	3 (4.8)
Sarcomatoid, *n* (%)	1 (1.6)
Pathologic stage	
pT1a, *n* (%)	26 (23.5)
pT1b, *n* (%)	10 (15.9)
pT2a, *n* (%)	4 (6.3)
pT2b, *n* (%)	2 (3.2)
pT3a, *n* (%)	20 (31.7)
pT3b, *n* (%)	1 (15.9)
Metastasis, *n* (%)	10 (15.9)
Follow‐up in months, mean (range)	77 (2–225)
Number of patients deceased at the time of analysis, *n* (%)	28 (44%)

### Pathologic Features

**Figure 1:**
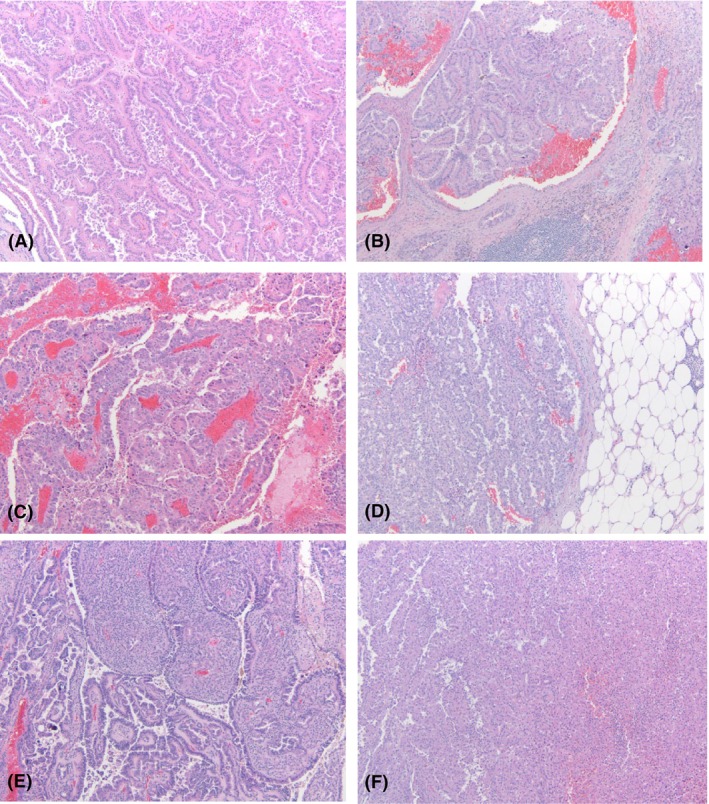
Representative histologic images of lesions selected for sequencing and found to harbour an (**A**) *ARID1A* and *STAG2* mutation, (**B**) *TERT* promoter mutation, (**C**) *SETD2* and *PBRM1* mutation, (**D**) *TP53* and *PBRM1* mutation, (**E**) *KMT2C* mutation and (**F**) *KDM6A* mutation.

The tumour size measured at gross examination ranged from 1.5 to 21.8 cm (mean—5.6 cm). The American Joint Committee on Cancer (AJCC) pathological T categories included cases classified as pT1a (*n* = 26), pT1b (*n* = 10), pT2a (*n* = 4), pT2b (*n* = 2), pT3a (*n* = 20) and pT3b (*n* = 1). At the time of diagnosis, metastases were seen in 9 cases to lymph nodes (hilar, paraaortic, paracaval, pericaval or retroperitoneal) and one case had metastases to the left arm soft tissue (Table 1). Histologically, the vast majority of cases had a papillary pattern (*n* = 59). There were three cases that had a mixed solid and papillary pattern and one case that had a sarcomatoid architectural pattern. The cases were composed of large cells with eosinophilic cytoplasm, pseudostratified or apically oriented nuclei and prominent nucleoli with high‐grade nuclear features seen in 30 of the cases (Figure 1 and Table 1). None of the tumours had a specific histology predicting their molecular makeup.

### Next‐Generation Sequencing

**Figure 2:**
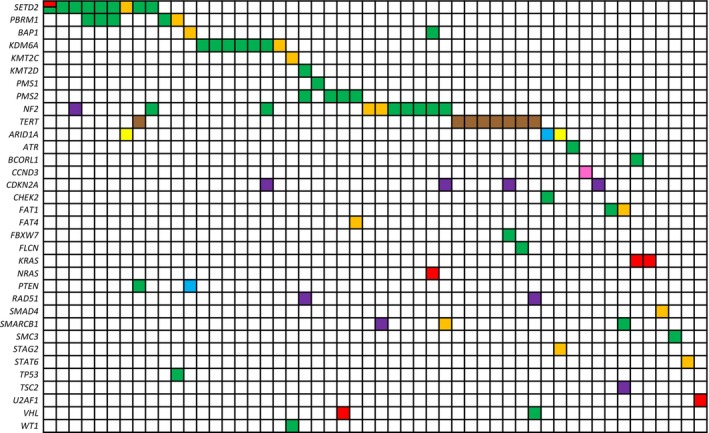
Summary of somatic mutations identified. Mutational landscape of recurrent somatic mutations based on UCM‐Oncoplus Assay. Mutated genes are listed on the left. Red—missense mutation; orange—nonsense mutation; yellow—in‐frame deletion or duplication; green—frameshift; blue—splice site mutation; purple—loss; pink—amplification; brown—promoter mutation.

NGS studies demonstrated that there was significant heterogeneity with these cases for pathogenic variants (Figure 2 and Table S1) and copy number plots (data not shown). The most commonly mutated genes were *SETD2* (*n* = 9), *PBRM1* (*n* = 5), *KDM6A* (*n* = 7), *PMS2* (*n* = 4), *NF2* (*n* = 7) and *TERT* (*n* = 7). We found pathogenic alterations in several other genes: *BAP1, KMT2C, KMT2D, PMS1, PMS2, ARID1A, CHEK2, ATR, CCND3, FAT1, FAT4, FBXW7, FLCN, KRAS, NRAS, PTEN, SMAD4, SMARCB1, SMC3, STAG2, STAT6, TP53, U2AF1, VHL* and *WT1*. Most of the pathogenic alterations were either frameshift mutations or nonsense mutations in tumour suppressor genes. Pathogenic copy number losses were noted in *NF2* (*n* = 1), *CDKN2A* (*n* = 4), *RAD51* (*n* = 1), *SMARCB1* (*n* = 1) and *TSC2* (*n* = 1) (Figure 2). No significant recurrent chromosomal alterations were identified. Of note, 10 of the cases were not found to have any pathogenic alterations in this study. Follow‐up RNA sequencing of these 10 cases demonstrated that these cases were negative for fusions (data not shown).

### Follow‐Up

The follow‐ups for the patients ranged from 2 months to 225 months (mean—77 months) with four patients being lost to follow‐up. At the time of analysis, overall survival showed 35 patients were alive and 28 patients were deceased (Table 1).

## Discussion

Histologic features of the included cases include papillary, tubulopapillary and solid architecture with cells usually containing abundant eosinophilic cytoplasm. The nuclei are large with prominent nucleoli, and the nuclei can be pseudostratified or monolayered with occasional inverted apical location.^15^ Notably, none of the tumours in our cohort had a specific histology predicting their molecular makeup. IHC studies have shown that these tumours are usually positive for P504S and CD10 (apical staining) with variable staining for CK7 and vimentin.^16^ Advances in molecular studies have shown that within this group, there is a wide range of underlying mutations, and several distinct and separate entities have been recognized by a new category in the 2022 WHO of ‘molecularly defined renal carcinomas’: *TFE3/TFEB* rearranged/altered RCC, *ELOC* (formerly known as *TCEB1*) mutated RCC, *FH*‐deficient RCC, succinate dehydrogenase‐deficient RCC, *ALK* rearranged RCC and *SMARCB1*‐deficient medullary RCC.^4^ However, these new subcategories only encompass a portion of the cases, and there continues to be ongoing debate about how to classify RCCs that exhibit papillary architecture and eosinophilic cytoplasm given the heterogeneity of histologic, IHC and molecular findings.^17^

Molecular studies have aided an improved delineation of pRCC, and we sought to reassess cases at our institution that had been previously diagnosed as pRCC, Type 2 or high‐grade pRCC. Our investigations revealed a wide range of genes that were mutated, with the most common ones being *SETD2* (*n* = 9), *PBRM1* (*n* = 5), *KDM6A* (*n* = 7), *PMS2* (*n* = 4), *NF2* (*n* = 7) and *TERT* (*n* = 7). Other studies characterizing the molecular alterations in pRCCs have identified similar alterations. Data from The Cancer Genome Atlas Research Network found recurrent mutations in *SETD2*, *NF2*, *PBRM1, BAP1, KDM6A, NFE2L2, FAT1* and *TP53*.^13^ Another smaller investigation following the genomic and epigenomic evolution of pRCCs identified mutations in *SETD2, PBRM1, NF2, SMARCB1* and *TERT*.^18^ Overall, the data support recurrent alterations in the Hippo signalling pathway (*NF2*), SWI/SNF complex (*SMARCB1* and *PBRM1*) and chromatin modifier pathways (*SETD2, KDM6A* and *BAP1*). Our findings support the heterogeneity of pRCCs with eosinophilic cytoplasm by further identifying cases that had molecular mutations in the RTK/RAS pathway (*KRAS* and *NRAS*), PI3K pathway (*TSC2*), TP53 pathway (*TP53* and *CHEK2*) and copy number alterations in the cell cycle pathway (*CDKN2A* and *CCND3*).^19^ Notably, two cases in our cohort had *KRAS* mutations and would now be characterized as papillary renal neoplasm with reverse nuclear polarity (PRNRP). These cases have been recently published in a series of renal tumours with RTK/RAS pathway alterations,^20^ but PRNRP is not recognized as a distinct entity in the most recent WHO classification.

Molecular studies of different renal neoplasms have highlighted that many are characterized by recurrent chromosomal alterations. Renal oncocytoma is characterized by recurrent loss of chromosomes 1 and X/Y^21^; ccRCC is frequently associated with chromosome 3p deletion^22^; and CHRCC has numerous chromosomal losses (chromosomes 1, 2, 6, 10, 13, 17 and 21).^11^ Studies of pRCC that were historically classified as Type 1 or lower grade have found gains of chromosomes 2, 3, 7, 12, 16 and 17 in ones.^13^, ^23^, ^24^ Other subgroups, including ones historically classified as Type 2, had fewer copy number alterations, although one subset of these has been identified with multiple chromosome losses, particularly chromosome 9p^13^. We did not identify any recurrent chromosomal alterations in our cohort, likely given the heterogeneity of pathogenic findings and the limited number of cases for each gene group.

Several studies that relied on the traditional characterization of pRCC, Type 1 and pRCC, Type 2 demonstrated that pRCC, Type 2 had an elevated all‐cause mortality and was associated with worse recurrence‐free survival when compared to pRCC, Type 1.^25^ Significant conclusions about different molecular alterations and prognostic outcomes were limited by the small sample size of this study. However, a general trend that was noted when examining the cases grouped by specific mutations is that 4 out of 6 patients (67%) with *KDM6A* mutations were deceased and 5 out of 8 patients (63%) with *NF2* mutations were deceased at the time of analysis. Studies looking at loss of *KDM6A* in various tumour types including pancreatic adenocarcinoma,^26^ acute myeloid leukaemia,^27^ bladder cancer^28^ and other tumour types^29^ have found that it characterizes a poor prognostic subtype. Previous analysis of RCC cases with *NF2* mutations demonstrated that while these cases did not have specific histologic features, desmoplastic stroma, fibrosis and calcifications could be commonly found; in addition, these cases had an aggressive disease course with frequent metastasis.^30^

Treatment options for patients with pRCC have been limited compared to ccRCC. Advances in therapies for pRCCs have lagged partly due to the heterogeneous nature of these lesions as well as a lack of pRCC‐specific randomized Phase III trials. Several different therapies have been investigated, including mTOR inhibitors, VEGF inhibitors, MET inhibitors, immunotherapy, and combination therapy.^31^ While targeted therapies are being developed for many of the genes in which pathogenic alterations were identified in our cohort, they are currently being investigated or approved in other tumour types. Some examples include therapies targeting *KRAS*,^32^ *NRAS*,^33^ the Hippo pathway^34^, ^35^ and *CDKN2A* loss.^36^ Improved characterization of the molecular landscape of RCCs with papillary architecture and eosinophilic cytoplasm may be important in identifying predictive biomarkers and selecting targeted therapies that can improve the otherwise dismal outcome of individuals with advanced disease.

## Funding information

This research received no specific grant from any funding agency in the public, commercial or not‐for‐profit sectors.

## Conflicts of interest

The authors have declared that no relevant financial conflicts of interest exist.

## Supporting information


**Table S1.** Specific molecular alterations identified including the gene, coding effect, c. nomenclature and p. nomenclature.

## Data Availability

The authors confirm that the data supporting the findings of this study are available within the article.
